# Cutaneous Squamous Cell Carcinoma of the Head and Neck

**DOI:** 10.1155/2011/502723

**Published:** 2011-02-21

**Authors:** Vivek V. Gurudutt, Eric M. Genden

**Affiliations:** Department of Otolaryngology–Head and Neck Surgery, Mount Sinai School of Medicine, 5 East 98th Street, 8th Floor, New York, NY 10029, USA

## Abstract

Cutaneous squamous cell carcinoma of the head and neck is an epidemic that reaches all parts of the world. Making the diagnosis relies on the acumen of the clinician and pathologist. Various pathologic subtypes exist and differ in histology and prognosis. High-risk tumors need aggressive treatment and vigilant surveillance to monitor for recurrence. Large tumors, deep tissue invasion, perineural involvement, recurrence, location in high-risk areas, and immunosuppression are implicated in worsening prognosis. Surgery is the mainstay of treatment with adjuvant radiation therapy as needed for aggressive tumors; however, other modalities are potentially useful for low-risk lesions. The use of Mohs surgery has become increasingly useful and has shown high success rates. Involvement of parotid and neck lymph nodes significantly affects outcomes and the physician should be comfortable with management of this complex disease. This paper examines the diagnosis, pathology, clinical course, and treatment options for cutaneous squamous cell carcinoma of the head and neck.

## 1. Introduction

Nonmelanoma skin cancer (NMSC) is the most common type of cancer. The American Cancer Society estimates an incidence of 1 million new cases per year in the United States (US) [1]. Medicare fee for service procedures for skin cancer increased 77% from 1992–2006 with 1.18 million patients being affected [2]. Extrapolation across the US population using medicare data brings the total number of patients treated for NMSC to over 2.1 million [2]. Cutaneous squamous cell carcinoma (cSCC) is the second most common skin malignancy in the US after basal cell carcinoma (BCC). The annual incidence of cSCC is from 200,000 to 300,000 per year and constitute 20% of nonmelanoma skin cancers [1]. Two thousand people die from NMSC each year in the US [1]. Sun exposed areas are at risk for development of disease with the head and neck having the highest incidence [3]. Increasing rates of disease could be related to increased sun exposure, ozone depletion, increasing length of life, and changes in clothing style allowing more exposure [4–6]. Highest rates of disease are noted among white populations with fair complexion, blue eyes, blonde or red hair, and in those that burn or freckle easily with sun exposure [5]. The majority of patients are men and the average age of onset is 66 [3]. The economic burden of skin cancer exceeds twenty-nine billion dollars in direct medical costs alone with over ten billion dollars lost to productivity opportunity cost [7]. The common nature of the disease requires clinicians to remain vigilant in surveillance and diagnosis. In this paper of cSCC of the head and neck, risk factors, pathology, diagnostic work up, and treatment are discussed.

## 2. Risk Factors

The most significant contribution to cSCC is from ultraviolet radiation (UVR) exposure. Although sunlight encompasses several wavelengths, ultraviolet A and B light are the most harmful to skin [5, 6, 8]. UVB light (200–320 nm) is more carcinogenic than UVA light (320–400 nm), although both can lead to malignancy [8]. In addition, the incidence increases with age and cumulative sun exposure [5, 6, 8, 9]. Ultraviolet exposure causes pyrimidine dimer formation that leads to point mutations in DNA and begins a cascade toward tumor formation [6, 8–12]. UVR can alter signal transduction within cells and also affect DNA repair mechanisms [10, 11]. The tumor-suppressor gene p53 has been implicated in tumor generation as well. UVR can alter gene activity in keratinocytes, allowing genetically altered cells to avoid apoptosis [10, 13]. This transcription factor can be inactivated by disruptions in DNA repair or single nucleotide mutations [10]. Oncogen activation of the ras pathway may also be implicated in cSCC formation [10, 14]. Furthermore, UVR acts as an immunosuppressant in the skin and further deranges the normal defense against malignancy [15]. Congenital diseases, such as xeroderma pigmentosum and oculocutaneous albinism, are autosomal recessive diseases with increased incidences of skin malignancy. Defects in DNA repair in the former and the lack of melanin in the latter leave patients of both disease processes susceptible to UVR-induced carcinogenesis [6]. Xeroderma pigmentosum carries a 2000-fold increased risk for cSCC [16].

Immunocompetence is vital in early cancer detection and destruction. Organ transplant recipients (OTRs) have an increased risk for development of skin cancers compared to the normal population. Immunosuppression is an independent predictor of survival [17, 18]. Renal transplant patients were noted to have 18 to 36 times the frequency of cSCC compared to the general public [9, 15]. In addition, the rate of cSCC becomes greater than that of BCC—reverse that of the normal population [19, 20]. An Irish study noted 50% of skin cancers in OTRs were cSCC compared to 25% that were BCC. The risk for cSCC in OTRs was 82 times greater than in the general population [20]. This phenomenon may be related to differences in major histocompatibility complex—1 between the two types of cancer [21]. Immunocompromised patients treated for metastatic cSCC to the parotid and neck have an increased risk of recurrence both locally and distally posttreatment with markedly decreased survival compared to immunocompetent individuals [22]. Southwell et al. describe a 0% two-year survival rate in immune-deficient patients compared to an 87% survival in normal patients [22]. Organ transplant recipients have an earlier age of onset of cSCC and a greater risk of developing deeply invasive or aggressive cSCC at the time of diagnosis [23]. In addition, Smith et al. noted increased acantholysis, angiogenic components, and tumor thickness histologically [23]. Recognition of the association of poor immune function with cutaneous malignancy has led to the creation of an international collaborative task force to formulate guidelines of immunosuppression reduction in transplant patients based on the severity of skin cancer development [24]. Patients with chronic lymphocytic leukemia or lymphoma are also noted to have increased incidence of cSCC [12, 25]. Mehramy et al. noted a recurrence rate 7 times that of controls in patients with chronic lymphocytic leukemia [25].

Viral diseases may also play a role in the development of NMSC. Individuals with human immunodeficiency virus are at high risk for developing aggressive cSCC, with an increased risk of recurrence and metastasis [26]. Human papilloma virus (HPV) has been implicated in the development of cSCC as well [1, 15, 27, 28]. Susceptibility to HPV is common in Epidermodysplasia verruciformis (EV) and has been associated with NMSC [28, 29]. Multiple subtypes compose the EV HPV group and are implicated in multiple skin conditions [28]. Incorporation of viral DNA into the genome of the cell or alteration of p53 is a potential mechanism of action [15]. 

Chronic inflammation, scars and wounds are another potential source of cSCC [5, 6, 8, 9, 12, 30]. No clear etiology from scarring or chronic wounds exists to explain the relationship with skin cancer. Possibilities include decreased immune surveillance and changes in local circulation [9]. A lack of early detection potentially explains the development of high-risk disease in such patients [12]. In the African American population, UVR plays a smaller role in cancer arising from scarred skin in sites outside the head and neck, and mortality from cSCC at these sites is greater than 18% [30]. 

Other risk factors include chemical exposures from tobacco, arsenic, and coal-tar products [5]. Ionizing radiation from occupational or therapeutic exposure can also lead to cSCC in a cumulative dose-dependent fashion [5, 6, 9].

## 3. Pathology

Multiple subtypes of cSCC exist. These subtypes differ histologically as well as prognostically. Cutaneous SCC originates from keratinocytes in the spinous layer of the epidermis. The most common form, or conventional type, has atypical keratinocytes invading the dermis. In addition, mitotic figures, hyperchromism, and pleomorphic nuclei are seen. Keratin pearl formation and intercellular bridging is common. This group of tumors can also be separated into well, moderately, and poorly differentiated forms [12, 16, 31]. The spindle cell variant is uncommon and occurs in UV damaged areas [32]. These tumors appear as ulcerated nodules or exophytic lesions and have a tendency toward perineural spread [12, 16, 32]. Furthermore, the spindle cell variant has a lack of differentiation and can behave aggressively while those not associated with radiation can be more indolent [31, 32]. Immunohistochemistry shows vimentin, cytokeratin, and epithelial membrane antigen positivity [33]. The verrucous variant of cSCC is a rare, low-grade disease presenting as a slowly enlarging, fungating lesion [32]. The pushing nature of the lower epithelium as broad projections into the dermis distinguishes this well-differentiated lesion [32]. Desmoplastic cSCC is an aggressive variant characterized by an invasive clinical course and poor prognosis. Patients have 10 times the risk of local recurrence and 6 times the risk of metastasis compared to other tumors [34]. The tumor has a pronounced stromal component with frequent perineural invasion and keratin pearls [31, 35]. Most cases are found on the ears, nose, and forehead [34].

## 4. Clinical Presentation

Cutaneous squamous cell carcinoma presents as an erythematous papule, plaque, or ulcer on a sun-exposed area of the skin that does not heal. They can be friable and may bleed with manipulation. They can be painful at times [37]. Verrucous variants present as raised, slowly enlarging wart-like lesions that may be locally invasive [37]. Actinic keratoses are precursor lesions that are scaly and vary in color and may involute spontaneously over time. Hyperpigmented papules can be seen in patients with bowenoid papulosis, while flat wart-like lesions are seen in epidermodysplasia verruciformis [37]. Bowen's disease is a form of in situ carcinoma with scaly lesions and erythema on sun-exposed areas [37]. In the head and neck, cSCC most commonly involves the ear, frontotemporal region, and cheek [38]. However, a recent study from Australia implicated the nose as the predominant site of disease [3].

## 5. Clinical Course

The extent of disease progression is dependent on the timing of diagnosis and treatment. Late detection may be complicated by more advanced disease and involvement of regional lymphatics. Lesions on the cheek, pinna, temple, forehead, anterior scalp, and postauricular area tend to metastasize to the parotid basin and level II lymph nodes [38, 40]. Tumors from the posterior scalp are more likely to travel to level V while more anterior parts of the face often bypass the parotid and involve levels I and III [38]. Analyzing the location of the lesion may help target lymph node basins for metastasis. Risk factors for regional metastasis include recurrence, size >2 cm, depth of invasion, involvement of deeper Clark levels, poor differentiation, perineural invasion, acantholysis, infiltrative strands of tumor, and lesions from existing scars [6, 8, 9, 12, 19, 41–43]. Moore et al. reviewed the course of 193 patients with cSCC of the head and neck and found that increased lymph node involvement also occurred when pathological analysis revealed lymphovascular invasion [42]. A retrospective review by Cherpelis et al. did not show increased metastatic spread associated with ulceration or inflammation [41]. Identifying high-risk lesions is vital to choosing an appropriate level of aggressiveness during treatment. Throughout the literature, high-risk cSCCs are recognized for their increased rates of recurrence and metastasis. Multiple studies cite factors that should be deemed high risk. The National Comprehensive Cancer Network (NCCN) guidelines provide a consensus list of factors to classify tumors as high risk [36]. Of note, the size criteria vary depending on location, which differs from numerous studies that describe high-risk lesions as greater than 2 cm in diameter [6, 8, 9, 12, 19, 37, 41–45]. A modified version of high-risk characteristics compiled by the NCCN is listed in Table 1. 

Traditionally, TNM staging for cSCC categorized lymph node metastasis as either positive or negative, irrespective of the involved nodal basins (parotid versus cervical). O'Brien et al. prospectively monitored 87 patients with parotid gland involvement. In their study, nodal disease was separated into parotid and cervical metastasis. Parotid nodes were separated into nodes <3 cm, nodes 3–6 cm/multiple nodal disease, or nodes >6 cm/facial nerve or skull base involvement and designated as P1, P2, or P3, respectively. Neck disease was staged as N0 for no neck disease, N1 for a single node up to 3 cm in size, and N2 for disease >3 cm or multiple or contralateral nodes [46]. Parotid staging revealed positive margins after parotidectomy increased as P stage increased. In addition, patients with N2 disease had decreased survival compared to patients with N0 and N1 disease [46]. The presence of disease in both the parotid and neck significantly decreased survival compared to parotid disease alone. As a result, O'Brien argued for P and N staging to better assess prognosis in patients with lymph node metastasis [46]. Several studies have also verified prognostic importance by separating nodal basin metastases [17, 18, 46–48]. In a study of 67 consecutive head and neck cSCC patients in New Zealand, Ch'ng et al. noted 37 patients with parotid nodal metastases. P staging was a significant independent prognostic indicator of survival (P3 survival < P1, P2) [18]. In addition, the combination of positive parotid and cervical nodes led to decreased survival compared to patients with involvement of only one of these basins [18]. Palme et al. retrospectively reviewed 126 patients with metastatic cSCC and found 81 patients with parotid disease, 45 patients with neck disease alone, and 14 patients with both. Increasing P stage negatively impacted prognosis in a significant fashion with 5-year disease-specific survival for P1, P2, and P3 being 81%, 51%, and 33%, respectively [17]. 

To better assess prognosis by clinical stage, Forest et al. have recently recommended changes to the P and N staging system as they do not feel that the staging groups have been adequately stratified or that the system adhered well when applied to pathological data [48]. Instead of separating parotid and cervical nodal metastases, Forest suggests including the groups together and stratifying based on number and size of nodes. This system, referred to as N1S3, classifies nodal metastases into three separate stages. The first stage includes patients with single lymph nodes up to 3 cm in size. Stage II encompasses single nodes >3 cm or multiple nodes up to 3 cm in size. Stage III patients have multiple lymph nodes greater than 3 cm [48]. The American Joint Commission on Cancer updated the staging system for cSCC in an effort to include more specific factors in prognosis. The AJCC system incorporates high-risk characteristics as well as a neck staging system similar to other head and neck malignancies. The AJCC system is listed in Table 2 [39]. Prognostic groups are included in Table 3 [39].

## 6. Diagnosis

Diagnosis of cSCC requires a total body skin examination. In addition, head and neck disease necessitates a complete head and neck physical examination. Suspicious lesions need biopsies that incorporate the depth of the lesion beyond superficial sampling. In areas that are close to vital structures or may impede cosmesis, incisional or punch biopsies may be taken. These biopsies should include the dermis and allow the presence of malignancy to be confirmed before a larger resection is performed. For smaller lesions away from critical areas, an excisional biopsy of the area allows for a diagnostic and potentially therapeutic procedure. If a lesion is suspicious for deeper invasion or regional metastasis, imaging may become necessary. CT scanning may be helpful in determining whether there is bony involvement, while magnetic resonance imaging is better for assessing nerve and potential dural spread.

## 7. Treatment

The mainstay of treatment for cSCC is surgical resection. However, other modalities of therapy are present throughout the literature. Fluorouracil (5-FU) is a topical chemotherapy agent that has approval in the United States by the Food and Drug Administration for treatment of actinic keratoses. It works by disrupting DNA synthesis and results in cell death. The use of 5-FU for cSCC is not recommended or widely utilized as a treatment for cancer [49]. Imiquimod is a synthetic agent used as an immune response modifier by stimulating cell surface receptors and increasing cytokine release [49]. Although its use in basal cell carcinoma is being studied, the appropriateness for cSCC is not supported and should not be used. Reports of off-label use for in situ disease outside of the head and neck have been reported with success and with further investigation may be found to be useful for noninvasive disease [50, 51]. 

Photodynamic therapy (PDT) is another treatment class that is being studied in the treatment of cSCC. PDT involves injecting a hematoporphyrin derivative into the bloodstream and treating with light therapy several hours after. The light is directed toward the area of malignancy or precancerous change to react with the photosensitizing agent to cause cell death with minimal disruption to normal skin [52]. In their guidelines for use of PDT for NMSC, Braathen et al. support the use of PDT for actinic keratosis and Bowen's disease as first line therapy. The group does not feel there is ample evidence to support its use for invasive cSCC [53]. An Austrian study noted a complete response in 54% of patients with superficial cSCC with a recurrence rate of 69% after a median followup of 8 months [54]. Schweitzer suggests use of photofrin-mediated PDT for elderly patients with large or multifocal recurrent lesions when surgical resection is contraindicated due to location or morbidity [52, 55]. 

Electrodesiccation and curettage can be used for small, superficial, well-defined lesions. This technique involves tumor removal down to the dermis and is practitioner dependent. Tumor extension into the subcutaneous fat requires a more formal surgical resection. Goldman describes a lower recurrence rate on the trunk and extremities secondary to a thicker dermis allowing better “feel” during curettage [56]. Werlinger et al. describe its use for cSCC, with 2 cases of recurrence after treatment of 56 patients compared to no recurrences with surgical excision. Average tumor size in the study was 7.9 mm [57]. Because recurrence rates are greater in higher risk tumors, electrodesiccation and curettage is reserved for small, superficial tumors in noncosmetically important areas [58]. Cryosurgery also can be used for low-risk, superficial disease. Curettage can precede cryotherapy without changes in effectiveness [59]. Kuflik describes the use of cryotherapy for NMSC over a 30-year period. In his study, most lesions ranged from 0.5 to 2 cm in size and had high cure rates [59]. Cryosurgery should not be used for high-risk tumors and has worse cosmetic outcomes compared to surgical excision [58, 60]. A major disadvantage for both cryosurgery as well as electrodesiccation and curettage is the lack of margin analysis during resection. 

The use of primary radiotherapy (RT) in patients with cSCC depends on multiple factors including cosmesis, function, age, medical morbidity, and patient desire [61, 62]. Disadvantages include cost, no histological control, and potential for side effects and secondary malignancy [61–63]. Local tumor control in small lesions rivals that of surgical resection, even in recurrent disease [61, 63]. However, as T stage increases, local control decreases [63]. In older patients with major cosmetic and functional disturbance with surgery and reconstruction, radiotherapy can be an attractive option. In addition, patients with unresectable disease may benefit from RT. Al-Othman et al. treated 88 T4 basal cell and squamous cell carcinomas using RT with curative intent. Local control was 53% at 5 years; however, including surgical salvage increased this figure to 90% [64]. Patients with tumor involving bone had lower survival rates or increased risk of bone complications [64]. In patients with regional metastasis, the use of surgery followed by RT is preferred to RT alone [17, 42, 47, 65–67]. Multiple studies have noted decreased disease-specific survival in patients treated with RT alone [65–67]. A University of Florida study found a 72% 5 year disease specific survival in patients treated with surgery and postoperative RT compared to 59% treated with RT alone [67]. In a separate study, 5-year local control rates were 83% with surgery followed by RT compared to 47% for RT alone [47]. Combined therapy allows for decreased recurrence rates and increased disease-free survival versus either RT or surgery alone [65]. Use of RT in patients with recurrence, perineural invasion, advanced disease, disease along embryonic fusion planes, and positive margins should be strongly considered [62, 68, 69]. Younger patients with a long life expectancy should avoid RT as a single modality because of the potential for late complications and decline in cosmesis over time [61]. 

Surgical resection with pathological monitoring remains the primary method of treatment for cSCC of the head and neck. Careful consideration of tumor margins, size of resection, as well as potential for reconstruction, functional deficit and cosmesis must be considered and thoroughly discussed before proceeding. Appropriate surgical margins for cSCC are 4 mm for low-risk lesions [70, 71]. This number is the minimal margin necessary to achieve greater than a 95% tumor clearance rate as determined by Brodland and Zitelli using Mohs micrographic surgery (MMS) [71]. A margin of 6 mm is suggested for higher-risk lesions [71]. The NCCN guidelines recommend 4–6 mm margins for low-risk tumors [36]. Larger margins, MMS, or complete circumferential peripheral and deep margin assessment with frozen section analysis is needed for higher risk lesions [36]. Brodland and Zitelli's criteria for high-risk lesions include tumor diameters >2 cm, higher histologic grade, subcutaneous tissue invasion, and location in high-risk areas (scalp, eyelids, ears, nose, and lips) [71]. Recurrence rates for conventional resection are reported in Rowe's review of treatment techniques as 8% over 5 years [19]. Griffiths et al. followed 93 patients after conventional resection and found 85 patients alive and disease free after 5 years resulting in a 91.4% disease specific survival [72]. Brantsch et al. used 3D-histology with surgical excision and followed 615 patients over an average of 43 months [73]. Local recurrence was 3% and depended on tumor thickness and amount of desmoplasia. A thickness of 2 mm or less, 2.1 to 6 mm, or >6 mm locally recurred in 0.5, 2.5, and 12.2% of patients, respectively [73]. Metastatic rate for tumors less than 2 cm was 1.9% versus a rate of 7.5% in lesions greater than 2 cm in size [73]. Metastasis-free survival over 6 years was 98% in patients with lesions less than 6 mm thickness without desmoplasia (low-risk). High-risk tumors with desmoplasia and thickness greater than 6 mm had a recurrence rate of 75% [73]. Again, the more high-risk the tumor, the greater the risk of recurrence and metastasis.

Surgical resection using Mohs micrographic surgery provides a high cure rate with an emphasis on tissue conservation [3]. The process involves serial sectioning of the tissue with comprehensive analysis of the tumor margins via horizontal frozen sections, allowing for greater assessment of the peripheral and deep surgical margins. Traditional vertical histologic sectioning techniques assess only a fraction of the margins to be visualized [3]. Sectioning of the tissue into quadrants and creating a tumor map with strict orientation to sectioning allows excellent assessment of lateral and deep margins. MMS allows better mapping than traditional excision, and repeat sectioning only occurs in areas with cancer to allow better tissue preservation. As a result, MMS is ideal for high-risk tumors and lesions in the cosmetically and functionally important areas of the eyes, nose, and lips. Preservation of form and function is essential when near the canthal apparatus of the eye, the oral commissure of the lip, and the ala of the nose. In tumors with deep extension, more invasive resection may become necessary to augment MMS. A prospective, multicenter case series in Australia over 10 years of MMS reviewed 1263 patients with squamous cell carcinoma. The technique had a 5-year recurrence rate of 3.9% [3]. Recurrent tumors had a higher recurrence rate of 5.9% compared to 2.6% of primary tumor excisions [3]. Recurrence rates in patients with perineural invasion were highest at 8% over 5 years [74]. Perineural invasion most often occurred in moderately to poorly differentiated tumors with larger tumor sizes, subclinical extension, and a higher number of MMS levels needed for excision [74]. Clinical perineural involvement had worse survival and control rates compared to those with incidental invasion found [3]. Because of the high cure rates and importance placed on tissue conservation, MMS remains an important tool in the treatment of high-risk cSCC [3, 12, 19, 62].

## 8. Treatment of the Parotid and Neck

Treatment of the lymph node basins draining cSCC primary sites is important to consider in high-risk, aggressive tumors. Various tumor sites require different levels to be included in selective neck dissection. Classically, tumors anterior to the external auditory canal require superficial parotidectomy and cervical neck levels II to IV, while posterior lesions require a posterolateral neck dissection in addition to levels II to IV. If the medial orbits, midface, or lips are involved, bilateral supraomohyoid neck dissections are needed [75–77]. Every cervical neck dissection should include the external jugular node as described by O'Brien, regardless of the site of lesion [40, 77]. Although elective neck dissections can be argued to be unnecessary since cSCC is a common tumor with uncommon neck disease, occult disease has been encountered [38]. Regional metastatic disease varies in the literature from 4% to 5.8% and has been noted to be as high as 20.7% [42, 73, 78]. The risk of metastasis to lymph nodes is related to risk factors such as increased tumor thickness, increased diameter, immunosuppression, arising from scars, and involvement of the ear [19, 73, 79]. Patients with lymphovascular invasion, poorly differentiated histology, perineural invasion, and subcutaneous invasion also have increased risk of metastasis [42]. Although controversial, elective neck dissection has a utility as a staging procedure and can help decision making regarding adjuvant therapy. In addition, for higher risk patient groups or tumors with extension into soft tissue, elective neck dissection can be considered [80]. Fine needle aspiration of suspicious nodes can be helpful to determine the need for neck dissection [36]. To better assess lymph node involvement, lymphatic mapping with sentinel lymph node biopsy (SLNB) is being investigated [81, 82]. Civantos et al. performed SLNB in patients that met high-risk criteria for cSCC. Their criteria included size >2 cm; evidence of deep invasion; immunosuppression; ear, lip, nasal vestibular location or tumor arising from a scar; high grade pathologic features; or rapid growth [82]. The negative predictive value was 98% and the false negative rate was 17% for skin disease [82]. Altinyollar studied 20 patients with lower lip lesions greater than 2 cm in size without palpable nodal disease. SLNB revealed no false positives, and disease was found in 16.6% of patients [83]. Renzi et al. noted all positive disease found with SLNB in their case series and those reviewed from the literature to have a size greater than 2 cm [84]. Most studies have small numbers and have varying definitions of high-risk patients necessitating SLNB. Furthermore, prospective multi-institutional studies are needed to verify the independent effect of specific risk factors for metastasis [80, 84, 85]. Use of SLNB for cSCC may be limited due to less metastatic spread compared to melanoma [38]. Further study of SLNB is necessary before its universal application in the management of high-risk cSCC [86]. 

 Clinical nodal disease in the parotid and neck should be addressed therapeutically. Metastatic disease affects the parotid gland in 60% to 82% of those patients with nodal spread [38, 87]. Cervical neck node disease without parotid involvement can be seen in 18 to 41% of patients [38, 87, 88]. In patients with positive parotid disease, spread to the cervical nodes can be occult, ranging from 16 to 42%, illustrating the importance of management of cervical nodes even if clinically negative [42, 66, 87]. Regional management of potential occult cervical nodes in those with known parotid disease is highly recommended.

Patients with positive lymph node disease have recurrence rates posttreatment ranging from 28% to 33% [46, 66, 87, 88]. Moore et al. examined 167 patients with nodal disease secondary to cSCC. Patients with combined surgery and RT had a 5-year local recurrence rate of 20% compared to 43% of patients with surgery alone. Disease-free survival at 5 years was also improved with combined therapy at a rate of 73% versus 54% of those with single modality therapy. Median time to recurrence was 8 months with 73% of locoregional failures eventually dying of disease [87]. Postoperative radiation is recommended in patients with regional disease. In a study of 74 patients with regional disease only to cervical nodes, Veness et al. found a 34% recurrence rate in a median time of 5.2 months with 4% being distant metastases [65]. In this study, disease specific survival was significantly higher in those receiving surgery and radiation compared to surgery or radiation treatment alone [65]. Andruchow et al. reviewed 322 patients with cSCC and lymph node metastasis with 90% treated with surgery and RT. Of the 105 recurrences, 42 occurred in the parotid, 33 presented in the neck, and 30 were distant metastases [88]. The importance of continued surveillance posttreatment of nodal disease is vital despite the advised combined modality therapy with surgery and radiation. 

Conflicting data in the literature exists in regards to the prognostic difference between parotid node and cervical node disease. Positive disease in both parotid and cervical nodes has shown worse prognosis than individual areas being affected in some studies [18, 88]. However, Palme et al. have shown similar survival rates regardless of location of disease and Moore et al. noted no difference in recurrence rates [17, 87]. The N1S3 staging system suggests that the presence of regional disease alone is the most critical factor, regardless of location in the parotid or cervical basins [48]. Location also does not alter how multiple nodal disease, nodal size 3 cm or greater, and extracapsular spread all decrease survival [18, 46, 48, 65].

The presence of high recurrence rates with high-risk disease and nodal metastasis has increased the need for further avenues of therapy. The addition of chemotherapy to surgery and RT is now under evaluation [89]. NCCN guidelines recommend combined radiation and cisplatin for incompletely excised nodal disease and possible use in those with extracapsular nodal extension [36]. Evidence for use is based on results from mucosal squamous cell carcinoma of the head and neck and not from prospective, randomized trials specifically for cSCC [36, 90–92]. Use of 5-fluorouracil, doxorubicin, or bleomycin added to cisplatin can be used in cases of regional recurrences or distant metastases, but, has little supporting data [36]. Additionally, case reports of cetuximab as therapy have shown some potential for future use [93–95]. This agent is a monoclonal antibody targeting epidermal growth factor receptor (EGFR). The increased expression or dysregulation of EGFR in NMSC makes use of cetuximab an attractive avenue of research for treatment of cSCC [96, 97]. Anti-insulin-like growth factor antibody with anti-EGFR antibody has shown induced apoptosis in vitro and improved survival in mice with cSCC [98]. Future avenues of therapy with monoclonal antibodies are becoming increasingly more studied for use in cSCC.

## 9. Followup

Patient followup for local disease includes visits every 3–6 months for 2 years, 6–12 months for 3 years, and annually thereafter [36]. Patients with regional disease or from high risk groups require followup every 1–3 months during year 1, every 2–4 months during year 2, every 3–6 months during years 3–5, and finally every 6–12 months thereafter. Certain high-risk populations (immunosuppression, OTRs, xeroderma pigmentosum, etc.) necessitate titrating examinations to disease frequency. All patients should be educated regarding sun protection and self-examination [36].

## 10. Conclusion

Treatment of cutaneous squamous cell carcinoma require the physician to be familiar with the characteristics of high-risk tumors and patient groups. Surgical resection remains the treatment of choice and MMS is especially useful in high-risk patients or in functional and cosmetic areas. Aggressive tumors metastasizing to the neck requires consideration of multimodal therapy with surgery and radiation. Chemotherapy is potentially useful in cases of extracapsular extension or in residual neck disease. Close surveillance, especially within the first two years after treatment, is recommended. Continued research is necessary to assess the utility of nonsurgical modalities of treatment as well as the use of sentinel lymph node sampling. Anti-EGFR antibodies have shown effectiveness with other cancers of the head and neck and have potential in treating cSCC in the future.

## Figures and Tables

**Table 1:** Characteristics of high-risk tumors. Adapted from NCCN Practice Guidelines in Oncology: Basal Cell and Squamous Cell Skin Cancers [36].

Size/Location:	
(a) 10 mm (cheeks, forehead, scalp, neck)	
(b) 6 mm^3^ (central face, eyelids, eyebrows, nose, lips, chin, ear, temple)	

Poorly defined borders	
Recurrent disease	
Immunosuppression	
Rapid Growth	
Site of prior radiation, scar	
Neurological symptoms	

Pathologic Criteria:	
(a)Moderately or poorly differentiated	
(b) Adenoid (acantholytic), adenosquamous, or desmoplastic	
(c) Depth:	
Clark level IV, V	
≥4 mm	
(d) Perineural or vascular invasion	

**Table 2:** AJCC TNM staging for cSCC [39].

*Primary tumor (T)*	
TX: Primary tumor cannot be assessed	
T0: No evidence of primary tumor	
Tis: Carcinoma in situ	
T1: Tumor 2 cm or less in greatest dimension with less than two high-risk features*	
T2: Tumor greater than 2 cm in greatest dimension or tumor any size with two or more high-risk features**	
T3: Tumor with invasion of maxilla, orbit, or temporal bone	
T4: Tumor with invasion of skeleton (axial or appendicular) or perineural invasion of skull base	

*Regional Lymph Nodes (N)*	
NX: Regional lymph nodes cannot be assessed	
N0: No regional lymph nodes	
N1: Metastasis in a single ipsilateral lymph node, 3 cm or less in greatest dimension	
N2a: Metastasis in a single ipsilateral lymph node, more than 3 cm but not more than 6 cm in greatest dimension	
N2b: Metastasis in multiple ipsilateral lymph nodes, none more than 6 cm in greatest dimension	
N2c: Metastasis in bilateral or contralateral lymph nodes, none more than 6 cm in greatest dimension	
N3: Metastasis in a lymph node, more than 6 cm in greatest dimension	

*Distant Metastasis (M)*	
M0: No distant metastasis	
M1: Distant Metastasis	

*Excludes cSCC of the eyelid.

**High-Risk Features for the Primary Tumor (T) staging: Depth/Invasion: >2 mm thickness, Clark level ≥IV; perineural invasion, Anatomic Location: Primary site ear, Primary site hair-bearing lip, and Differentiation: Poorly differentiated or undifferentiated.

**Table 3:** AJCC Prognostic groups based on TNM staging [39].

Group	T	N	M
0	Tis	N0	M0
I	T1	N0	M0
II	T2	N0	M0
III	T3	N0	M0
	T1	N1	M0
	T2	N1	M0
	T3	N1	M0
IV	T1	N2	M0
	T2	N2	M0
	T3	N2	M0
	T any	N3	M0
	T4	N any	M0
	T any	N any	M1
